# 

**Published:** 1823-12-01

**Authors:** 


### BIBLIOGRAPHICAL RECORD;

OR,

*Books received for Review since last Quarter.*

1. Transactions of the Associated Apothecaries and Surgeon-Apothecaries of England and Wales. Volume 1, pp. 170 Preface, and 424 Text. Burgess and Hill, October, 1823.

---

2. Medico-Chirurgical Transactions. Vol. 12, Part. II pp. 300, with plates.

---

3. First Steps to Botany, intended as Popular Illustrations of the Science, leading to its Study, as a Branch of General Education. By James L. Drummond, M.D. Professor of Anatomy and Phisiology in the Belfast Academical Institution. pp. 389, with numerous wood-cuts.

☞ *This little work is written in a very attractive style, and is well calculated to answer the object which the ingenious and classical author had in view.*

---

3. A Treatise on Midwifery; developing new Principles, which tend materially to lessen the Sufferings of the Patient, and shorten the Duration of the Labour. Second Edition, considerably improved, and illustrated with numerous Cases and additional Observations, &c. By John Power, M.D. Physician Accoucheur to the New Westminster Lying-in-Charity, &c. 8vo. pp. 234, London, October, 1823.

---

4. The American Medical Recorder, of Original Papers and Intelligence in Medicine and Surgery, No. 23, for July, 1823. (In Exchange.)

☞ *In this Number, the Publisher has liberally offered a premium of* 100 *dollars for the best Essay on the prevailing Epidemic Fevers of the United States.*

---

5. Revue Medicale, for June and July, 1823.

---

6. Postcript to Mr. Moir's Notes on Dr. Macintosh's Treatise on Puerperal Fever, pp. 22.

☞ *Sincerely do we hope that this may conclude the war of words going forward among our northern brethren. No party can win, and all may lose at this unprofitable game.*

7. Dissertatio Medica Inauguralis de Synocho. AUCTORE JACOBO WRIGHT, M.D.

☞ *Contains many judicious remarks and ingenious observations.*

---

8. Archiv fur Medizininsche Erfahrung, &c. for January and February, March and April, 1823.

☞ *We return Dr. Wagner many thanks for the above numbers.*

---

9. Recherches sur le Remollissement du Cerveau; Ouvrage dans le quel on s'efforce de distinguer les Diverses Affections de ce Viscère par des Signes Caracteristiques. Par LEON ROSTAN, Medecin de l'Hospice de la Veillesse-femmes, Professeur de Medecine Clinique. *Seconde Edition,* pp. 503. Paris, 1823.

---

10. De la Medecine Operatoire, par R. B. SABATIER. Nouvelle Edition faite sous les Yeux de M. LE BARON DUPUYTREN, par L. S. SANSON, M.D. et L. J. BEGIN, Chirurgen, &c. In two volumes, 8vo. Paris, 1822.

---

11. Clinique Medicale, ou Choix d'Observations recueillies a la Clinique de M. LERMINIER, par G. ANDRAL, FILS, M.D. Premier Partie Fievres.

---

12. A popular Treatise on Accidents, &c. for the Use of Persons residing at a distance from Medical Assistance. 8vo. pp. 107. Glasgow, 1823.

---

13. Observations and Commentaries on Medicine, &c. &c. &c. By ADAM DODS, M.D. &c. &c. 8vo. pp. 82. Worcester, 1823.

---

15. Travels in Ireland, in the Year 1822, exhibiting brief Sketches of the Moral, Physical, and Political State of the Country: with Reflections on the best Means of Improving its Condition. By THOMAS REID, Member of the Royal College of Surgeons in London, and Surgeon in the Royal Navy. 8vo. 1823.

---

16. Two Voyages to New South Wales and Van Dieman's Land, with a Description of the Present Condition of that Interesting Colony; including Facts and Observations relative to the State and Management of Convicts of both Sexes; and also Reflections on Seduction and its General Consequences. By THOMAS REID, Member of the Royal College of Surgeons, &c. 8vo. 1822.

---

17. Formulary for the Preparation and Mode of Employing Several of the New Remedies, namely, Nux Vomica, Morphine, Cinchonine, &c. with an Introduction and copious Notes. By CHARLES THOMAS HADEN, Surgeon to the Chelsea and Brompton Dispensary, &c. Translated from the French of F. MAGENDIE, 3d Edition, 8vo. pp. 112. Underwoods, October, 1823.

☞ *This interesting little work in our next.*

18. A Practical Treatise on Tropical Dysentery, more especially as it occurs in the East Indies; illustrated by Cases and Appearances on Dissection. To which are added Practical Treatises on Scorbutic Dysentery, on the Morbus Chylopoieticus and Gastrodynia a Fame; with some Facts and Observations relative to Scurvy in General, and a short Account of the Scorbutic Disease that appeared at the Penitentiary, Milbank. By R. W. BAMPFIELD, Esq. Surgeon. Author of an Essay on Hemeralopia, &c. formerly Surgeon to the Belliqueux and Warrior; and now one of the Surgeons to the Royal Metropolitan Infirmary for Diseases of Children, 8vo. Second Edition, pp. 344. London, 1823.

☞ *This highly valuable work, a review of which we gave in one of our early numbers, is now republished with many additions and improvements. It forms an indispensible portion of the medical library of every practitioner proceeding to the East or West Indies.*

---

19. A System of Anatomical Plates; accompanied with Descriptions, and Physiological and Pathological Observations. By JOHN LIZARS, F.R.S. &c. Part II. and III. Blood-vessels and Nerves. Edinburgh and London, 1823.

☞ *We are happy to see that the patronage of the public, on both sides of the Tweed, is liberally extended to this very deserving work. The plates increase in beauty and fidelity, while the letter press is a model of precision and perspicuity.*

---

20. Recherches Experimentales sur les Fonctions du Systême Nerveux. 1ier. Memoire de l'Influence du Systême Nerveux sur la Digestion. Par M. M. BRESCHET, EDWARDS, et VAVASSEUR. Paris, 1823.

---

21. Observations on Fractures of the Neck of the Thigh-bone, being an Appendix (the 2d) to the Work on Dislocations and Fractures of the Joints. By SIR ASTLEY COOPER, Bart. Surgeon to the King, &c. Quarto, pp. 44, and three plates, 1823.

---

22. De Sede et Caussis Vesaniæ. AUCTOR FRANCISCUS FRANCKE, M.D. Lipsiæ, 1823.

☞ *We return Dr. Francke many thanks for this ingenious thesis and the polite letter accompanying it. We will attend to the recommendation he has given of his friend.*

---

23. Remarks on an Institution for the Preparation and Use of Artificial Mineral Waters in Great Britain. By DR. STRUVE of Dresden. To which are added, Testimonials, by DR. KREYSIG of Dresden, and Dr. CLARUS of Leipzig. 8vo. sewed, pp. 16. London, 1823.

☞ *For some years past, Dr. Struve, of Dresden, has excited much attention by his successful imitation of some of the most celebrated mineral waters on the continent, especially those of Pyrmont, Selters, Spa, Carlsbad, Marienbad, &c. The Testimonies of Drs. Kreysig and Clarus, as to the perfect similarity of these Artificial with the Native Waters, and the identity of their Physical effects, are very satisfactory. An apparatus and manufactory of these are now about to be erected in this country, probably at Brighton, by two gentlemen from Dresden, and they will therefore be put to the test of experience. We shall give further intelligence as it may reach us.*

24. Suggestions on the Use of Mercurial Friction on and under the Right Breast, to palliate the Evils of the Fever of Hot Countries. By William Forbes, A.M. Surgeon. Manchester, Jamaica.—8vo. sewed.

☞ *This is a poor production.*

---

25. The Lancet, No. 6, for Sunday, November 9, 1823. pp. 36, price 6d.

☞ *We hold it to be a breach of etiquette for one periodical publication to animadvert on the conduct of another, unless attacked or interfered with itself. We mean, of course,* medical *journals ; for political publications use no ceremony with each other. In offering a word or two of advice to our young friend the* Lancet, *and through him to several other of our juvenile cotemporaries, we trust they will not impute to us any unworthy motives, and that, on mature reflection, they will acknowledge the propriety of the advice we give.*

*We are convinced then, that no medical periodical publication can succeed, unless conducted on* unexceptionable *principles—we mean, such principles as cannot* reasonably *be objected to by the members of the profession. We think that the unauthorized publication of a man's lectures, as they are delivered from day to day, cannot be defended on principles of morality, equity, or medical ethics ; and therefore we advise our junior cotemporaries not to take advantages of, or rely upon, a practice which can, at best, but afford a temporary interest. Secondly, we would dissuade them, if in our power, from mixing up politics, theatrical criticisms, and other heterogeneous matters, with medical science or literature. It is an unnatural union which cannot long hold together, and has invariably failed hitherto, in every instance where it was attempted.*

*The above exceptions made, we wish every success to the Lancet, and all other candidates for public favour—nor will we ever refuse to give whatever publicity they require through the medium of this Journal.*

---

26. A Statement of the Early Symptoms which lead to the Disease termed Water in the Brain; with Observations on the Necessity of a Watchful Attention to them, and on the fatal consequence of their Neglect. By G. D. Yeats, M.D. F.R.S. Fellow of the Royal College of Physicians of London, &c. &c. Second Edition, considerably enlarged, 8vo. pp. 164. London, 1823.

---

27. Observations illustrative of the History and Treatment of Chronic Debility, the Prolific Source of Indigestion, Spasmodic Diseases, and various Nervous Affections. By William Shearman, M.D. Member of the Royal College of Physicians, President of the Medical Society of London, Senior Physician to the Royal West London Infirmary, and Physician to the London Dispensary. 8vo. pp. 225. London, 1823.

---

### ERRATA.

396 *line* 18 *for* Etymoligies *read* Etymologies.
624 *line* 17 *for* Healing *read* Heating.
634 *line* 11 *(bottom) for* Elimmated *read* Eliminated.

The communications received from Mr. Shipman, Mr. Ridge, Mr. Harvey, &c. have been transmitted to our respected cotemporaries, the Medical and Physical Journal, and the Medical Repository, after publication, in which they will be duly noticed in our Periscope department.

